# CRYPTIC CHOICE OF CONSPECIFIC SPERM CONTROLLED BY THE IMPACT OF OVARIAN FLUID ON SPERM SWIMMING BEHAVIOR

**DOI:** 10.1111/evo.12208

**Published:** 2013-08-12

**Authors:** Sarah E Yeates, Sian E Diamond, Sigurd Einum, Brent C Emerson, William V Holt, Matthew J G Gage

**Affiliations:** 1 School of Biological Sciences, University of East AngliaNorwich Research Park, Norwich, NR4 7TJ, United Kingdom; 2 Centre for Biodiversity Dynamics, Department of Biology, Norwegian University of Science and Technology, NO-7491Trondheim, Norway; 3 Norwegian Institute for Nature Research, NO-7485Trondheim, Norway; 4 Island Ecology and Evolution Research Group (IPNA-CSIC), C/Astrofísico Francisco Sánchez 338206 La Laguna, Tenerife, Canary Islands, Spain; 5 Academic Department of Reproductive and Developmental Medicine, University of SheffieldLevel 4, Jessop Wing, Tree Root Walk, Sheffield, S10 2SF, United Kingdom

**Keywords:** Fertilization, gamete, salmon, sperm competition, trout

## Abstract

Despite evidence that variation in male–female reproductive compatibility exists in many fertilization systems, identifying mechanisms of cryptic female choice at the gamete level has been a challenge. Here, under risks of genetic incompatibility through hybridization, we show how salmon and trout eggs promote fertilization by conspecific sperm. Using in vitro fertilization experiments that replicate the gametic microenvironment, we find complete interfertility between both species. However, if either species’ ova were presented with equivalent numbers of both sperm types, conspecific sperm gained fertilization precedence. Surprisingly, the species’ identity of the eggs did not explain this cryptic female choice, which instead was primarily controlled by conspecific ovarian fluid, a semiviscous, protein-rich solution that bathes the eggs and is released at spawning. Video analyses revealed that ovarian fluid doubled sperm motile life span and straightened swimming trajectory, behaviors allowing chemoattraction up a concentration gradient. To confirm chemoattraction, cell migration tests through membranes containing pores that approximated to the egg micropyle showed that conspecific ovarian fluid attracted many more spermatozoa through the membrane, compared with heterospecific fluid or water. These combined findings together identify how cryptic female choice can evolve at the gamete level and promote reproductive isolation, mediated by a specific chemoattractive influence of ovarian fluid on sperm swimming behavior.

We now know that the stages between mating or gamete release and fertilization provide a wealth of opportunity for the evolution of cryptic processes that can have profound influences on individual reproductive success and gene flow (Eberhard 1996; Birkhead and Pizzari 2002; Birkhead et al. 2009; Howard et al. 2009). Opportunities for females to gain reproductive fitness improvements at this postmating, prezygotic stage might be widespread. For example, common garden experiments that can control for direct parental effects using external fertilizers, and where split-brood designs equalize maternal effects, demonstrate that there is substantial potential for females to improve their reproductive success if they can encourage those sperm conferring the highest offspring fitness to be the successful fertilizers (Wedekind et al. 2001; Rudolfsen et al. 2005; Evans et al. 2007; Pitcher and Neff 2007; Rodriguez-Munoz and Tregenza 2009). Alongside this evidence from external fertilization systems for variation in male:female compatibility, is the mounting evidence from internal fertilization systems for mechanisms at the gamete level, which allow females to promote fertilization by sperm from those males that will give the resulting offspring improved fitness (see meta-analysis in Slatyer et al. 2012), for example, when at risk of inbreeding and fertilization by close genetic relatives (Tregenza and Wedell 2002; Michalczyk et al. 2011).

Despite this convincing background for the existence of postmating sperm selection, clearly demonstrating how females or their eggs are able to “choose” sperm from particular males has lagged behind the indirect evidence for a number of important reasons (Birkhead 1998; Pitnick and Brown 2000). First, there must be a clear a priori basis for the existence of “right” and “wrong” sperm in the reproducing population, with established fitness consequences for fertilization by either. Second, the recognized influences of male-derived traits on fertilization success (such as sperm quality or quantity) should be isolated and ideally independent of the most “preferred” or “compatible” males. Finally, identifying the mechanism allowing cryptic female choice of sperm poses particular problems: creating unconfounded experimental control at the level of the gamete, within the intimate environment of the female reproductive tract, and while preserving normal sperm and egg interactions for objective measurement, all present obvious practical and technical hurdles. Because of these obstacles, proving exactly how females choose the “right” sperm for fertilization that will maximize offspring fitness has been a challenge (Birkhead 1998; Pitnick and Brown 2000).

One widespread situation, which obviously satisfies the first requirement that there be a clear a priori basis for the existence of compatible and incompatible sperm in the potential fertilization set, is where postmating risks of hybridization exist (Birkhead and Brillard 2007). These risks may become prevalent under a number of conditions that include the following: (1) when premating hybridization barriers are nonexistent, for example, in multispecies simultaneous broadcast spawning (Vacquier 1989); (2) where barriers are weak, for example, across Hybrid zones where speciation is currently in progress (Barton and Hewitt 1989); (3) where mating barriers are overridden by sexual conflict because high mating potential or low cost reduces the strength of selection in males to avoid hybrid matings (Parker and Partridge 1998); or (4) if hybridization is maintained because it is a form of interspecific competition within sympatry (Wolf et al. 2001). Under these conditions, conspecific sperm precedence (CSP, Howard 1999) can be an important enforcer of reproductive isolation (Coyne and Orr 2004), where mechanisms acting after mating, but before zygote formation, bias conspecific sperm and/or discourage heterospecific sperm to fertilize. CSP is “the favored utilization of sperm from conspecific males in fertilization when both conspecific and heterospecific males have inseminated a female” (Howard 1999). CSP can be symmetrical, where each potentially hybridizing species show equal fertilization incompatibility (e.g., Geyer and Palumbi 2005), or it can be asymmetrical, where incompatibility is most pronounced only in one crossing direction (e.g., Bella et al. 1992, Dean and Nachmann 2009).

Now we appreciate that postcopulatory mechanisms of competition and choice can have profound effects upon gene flow and reproductive success, CSP is becoming more widely recognized. CSP is now identified in fishes (e.g., *Etheostoma* darters, Mendelson et al. 2007), insects (e.g., *Chorthippus* grasshoppers, Bella et al. 1992; *Tribolium* beetles, Wade et al. 1994; *Drosophila* fruit flies, Price 1997; *Allonemobius* and *Gryllus* crickets, Howard and Gregory 1993 and Tyler et al. 2013), and broadcast spawning marine invertebrates (e.g., *Echinometra* urchins, Geyer and Palumbi 2005). These systems reveal that mechanisms operating at the level of the sperm and egg can play important roles in maintaining reproductive isolation between species and, with this background, biologists are now focusing efforts on the challenges of understanding how these mechanisms of sperm–egg interaction operate.

Despite the importance of sperm–egg interactions for gene flow (Howard 1999; Coyne and Orr 2004), we understand remarkably little about exactly how females encourage the “right” sperm to fertilize their eggs, when faced with the risk of fertilization by heterospecifics. An exception here is the broadcast spawning marine invertebrate model systems of urchins and abalone, where mechanisms of sperm–egg interaction are very well established. Because of the lack of precopulatory barriers to hybridization, broadcast spawning selects for sperm–egg interactions to avoid heterospecific sperm (Howard et al. 2009). Specific associations between bindin molecules in urchins (Palumbi 1999) and vitelline envelope receptor for lysin (VERL) and lysin in *Haliotis* (Swanson and Vacquier 2002) constrain heterospecific sperm attachment or egg membrane penetration, usually blocking hybridization at the gamete level (Metz et al. 1994; Palumbi 1999). Although these species-specific fertilization mechanisms are understood in impressive detail (Vacquier 1998; Lessios 2011), the parallel approach using sperm choice experiments that test for CSP “has rarely been tested explicitly for among broadcast spawners” (Palumbi 1999); one clear exception is the study of *Echinometra* urchins showing clearly symmetrical CSP (Geyer and Palumbi 2005). In the other systems employing sperm choice experiments and revealing CSP, mechanistic details lag behind the evidence for sperm selection. In *Drosophila*, the use of spermless males identified male seminal fluid as a key component controlling CSP (Price 1997; Price et al. 2000), and fewer heterospecific sperm were stored or showed motility in the female tracts and sperm storage organs of hybridizing *Epilachna* ladybirds or *Allonemobius* crickets (Katakura 1986 and Gregory and Howard 1994). In both *Callosobruchus* beetles and *Gryllus* crickets, where CSP exists, quantification of sperm in female storage revealed that fewer heterospecific sperm are stored when conspecific inseminations also take place; interestingly, these heterospecific sperm also showed significant fertilization disadvantages relative to their numerical representation in storage (Rugman-Jones and Eady 2007; Tyler et al. 2013), indicating that additional mechanisms of sperm selection operate somewhere between sperm storage and egg fertilization.

One of the key challenges to explicit identification of cryptic mechanisms of sperm choice is the practical and technical difficulties of measuring sperm–egg interactions at the intimate level of the gamete, using a controlled experimental approach. Invasion of the female tract to observe in vivo sperm behavior is both technically demanding, and likely to disrupt normal gamete or tract behavior, whereas observations of sperm activity on a microscope slide are not likely to be measuring behavior in the physical or chemical environment to which the gametes are adapted to function. In this study, we overcome these challenges by examining how females distinguish between sperm in an externally fertilizing system, where controlled fertilization experiments can be performed, and sperm behavior measured, in the microenvironment to which the gametes are naturally adapted, thereby providing meaningful measures of fertilization outcomes and sperm behavior under experimental control (Gage et al. 2004; Yeates 2005; Yeates et al. 2009). In addition, we can control directly for any absolute effects of differential fertility or competitiveness between individuals through the use of split-brood and split-“ejaculate” paired design in vitro fertilization and competition experiments (Yeates et al. 2009), thereby identifying fertilization outcomes resulting specifically from sperm–egg compatibility. Ultimately, we also study a reproductive system where the fitness costs of natural hybridization provide clear a priori expectations for the evolution of cryptic female choice of genetically compatible conspecific sperm to avoid outbreeding depression (Barton and Hewitt 1989; Garcia-Vazquez et al. 2002).

Congeneric Atlantic salmon (*Salmo salar*) and trout (*Salmo trutta*) exist and spawn in sympatry across much of their range. Although some spatial segregation exists across spawning areas, hybridization is generally avoided by a 15-day difference in peak spawning activity (Heggberget et al. 1988). However, natural hybridization does occur between these species (Verspoor and Hammar 1991), especially where river systems are disturbed by humans (Hindar and Balstad 1994). Premating barriers to hybridization in externally fertilizing fish species can be relatively weak and are widely documented (Verspoor and Hammar 1991), possibly exacerbated under multimale spawning conditions (Weir et al. 2010). Because male salmonids can be fertile for a much longer time window than females, and greater than 15 days (Yeates 2005), the potential for hybridization between salmon and trout within the same river systems is evident (Garcia de Leaniz and Verspoor 1989; Hindar and Balstad 1994). Despite the ease of generation of salmon–trout hybrids, which can be fertile (Garcia-Vazquez et al. 2004), they do not represent longer term prospects for successful introgression and have very different chromosome numbers (*S. trutta*: 2*n* = 80, *S. salar*: 2*n* = 58 [typically], Pegington and Rees 1967). Because of these significant reproductive costs of hybridization (Barton and Hewitt 1989; Garcia-Vazquez et al. 2002), selection is predicted to favor postmating female adaptations that avoid fertilization by genetically incompatible sperm (Coyne and Orr 2004). Because salmon and trout spawn externally, we were able to perform controlled in vitro fertilization and competition experiments that allowed us to measure patterns of sperm–egg association between and within these two species, and in the absence and presence of sperm competition. We first establish that both species are fully interfertile at the gamete level, even under limited sperm–egg association times, and then demonstrate that fertilization precedence is significantly biased if eggs are given a choice of sperm. We investigate whether ovarian fluid, a semiviscous liquid containing a complex of inorganic ions, sugars, proteins, hormones, and enzymes derived from secretory epithelia in the ovaries and filtered blood plasma (Lahnsteiner et al. 1995; Rosengrave et al. 2009), has an influence on fertilization dynamics and sperm behavior. The function of ovarian fluid is not yet fully understood within external fertilization, but it bathes the eggs in storage and is released at spawning (Lahnsteiner et al. 1995; Rosengrave et al. 2009). Importantly, ovarian fluid influences sperm swimming parameters in fish (Tuner and Montgomerie 2002; Rosengrave et al. 2009), either increasing (Butts et al. 2012) or slowing (Gasparini and Pilastro 2011) sperm movement according to male–female relatedness, so a role in cryptic female choice has been suggested (Rosengrave et al. 2008; Gasparini and Pilastro 2011). Ultimately, we isolate the factor that allows this cryptic fertilization choice by eggs, and how it acts on sperm behavior to explain the competitive success of conspecific sperm.

## Materials and Methods

### FIELD SITE AND FISH GROUPS

Fertilization trials and egg rearing were carried out at the Norwegian Institute of Nature Research (NINA) Aquatic Research Station in Ims, Norway, where fish were maintained and handled according to standard hatchery protocols approved by the Norwegian Animal Research Authority. Gametes for most experiments were recovered from fish that had been hatched and reared in the hatchery at Ims, and sourced from the nearby River Figgjo. Adult fish therefore experienced similar environmental backgrounds, and the hatchery rearing allowed close monitoring of multiple adults entering breeding condition so that we were able to source ripe males and females of both species for simultaneous in vitro fertilization and competition experiments. One exception was the sperm migration experiment that was conducted using wild caught salmon from the River Imsa, and wild caught trout from the nearby River Fossbekk. Fish were maintained and handled according to standard hatchery protocols approved by the Norwegian Animal Research Authority. Adult fish were kept as single species, mixed-sex adult groups in 4000 L tanks fed directly by natural River Imsa water. At the onset of the spawning season in October, adults were checked daily, and gametes stripped from fish showing full reproductive condition with free-running eggs or milt, using standard hatchery procedures (Gage et al. 2004; Yeates 2005; Yeates et al. 2009). Stripped gametes were stored before experimentation for a maximum of 5 days on wet ice just above 0°C in airtight, oxygenated bags. Our use throughout of reciprocally paired cross-fertilization designs, where focal males were compared in both “conspecific” and “heterospecific” conditions (see IN VITRO FERTILIZATION EXPERIMENTS below), enabled control for any directional effect of gamete storage on individual fertilization success (Yeates et al. 2009). Additional checks on sperm fertility after storage showed no change under these conditions: tests of average %fertility of 15 μl sperm-extender solutions (which create sperm-limiting conditions) on day of strip did not change after 5 days of oxygenated storage on ice (salmon: *t*_9_ = −0.05, *P* = 0.961; trout: *t*_7_ = 0.614, *P* = 0.558; data normally distributed Kolmogorov–Smirnov tests *P* > 0.06).

### IN VITRO FERTILIZATION EXPERIMENTS

#### General methods

Prior to use in fertilization trials and sperm competitions, milt subsamples were diluted in Trout Extender (80 MM NaCl, 40 mM KCl, 1 mM CaCl_2_, and 20 mM Tris, adjusted to pH 9, Yeates 2005) at a 1:1 ratio. This procedure reduces the risk of any preactivation of the sample, and predilutes the semiviscous milt so that sperm are simultaneously and evenly activated on contact with water (Yeates 2005). All in vitro fertilizations took place in dry 1 L plastic beakers, with egg batches placed on one side opposite to the sperm-extender sample. Fertilizations were conducted by introducing either 100 mL or 500 mL (depending on the experiment) of Imsa river water (at natural temperatures of 4–8°C), which activated and mixed the sperm and egg batch simultaneously. After all in vitro trials, fertilization solutions were left to stand for at least 3 min after gamete activation, by which time the fertilization is complete (Gage et al. 2004; Yeates 2005; Yeates et al. 2009). Egg batches were then allowed to develop in uniquely coded trays in incubation channels with constant river water flow at natural temperatures (Gage et al. 2004; Yeates 2005; Yeates et al. 2009).

#### Noncompetitive fertilization trials between salmon and trout gametes

Eggs and sperm were stripped from *n* = 15 female and male salmon and *n* = 15 female and male trout. For each female, two egg batches were created containing approximately 100 eggs (range 87–127), which were then fertilized using 200 μl sperm-extender solutions from either a salmon or a trout in 500 mL Imsa water. Thus, *n* = 15 pure and *n* = 15 hybrid in vitro crosses were created for both salmon and trout (*n* = 60 total fertilizations), which allowed replicated, pairwise comparisons of relative fertilization rates of salmon and trout females with either conspecific or heterospecific sperm. To score fertilization success, eggs were soaked in 5% acetic acid after 15 days of incubation, allowing visualization of developing embryos in fertilized eggs (Yeates 2005). Fertilization datasets did not all conform to normal distributions (Kolmogorov–Smirnov tests *P* < 0.05), so fertilization success of eggs exposed to conspecific versus heterospecific sperm was compared across *n* = 15 females using paired analyses on square root arcsine transformed data.

#### Fertilization rates in salmon and trout ovarian fluid under limited sperm exposure times

To determine whether ovarian fluid influences the dynamics of interfertility between salmon eggs and salmon and trout sperm, and whether this was affected by ovarian fluid under limited sperm–egg exposure times, we ran trials where we exposed salmon eggs to either salmon or trout sperm, in either salmon or trout ovarian fluid, and controlling the sperm–egg exposure times to either 2, 5, or 10 sec. Although gamete association in salmonids is rapid (Gage et al. 2004; Yeates et al. 2007), the 2 and 5 sec gamete exposure windows were designed to limit fertilization success, and thereby enable us to determine relative fertility of conspecific and heterospecific sperm, and whether the dynamics of this fertility was influenced by ovarian fluid. To separate eggs from their ovarian fluid identity, strips from ripe females were sieved just prior to fertilization trials, and ovarian fluid collected in a separate beaker. Eggs were then divided into smaller batches containing an average of 63 eggs (range 46–104), each held in a sieve and washed in isotonic solution (90 g NaCl in 10 L of Imsa river water) just prior to fertilizations to rinse away any remaining fluid from the surface of the eggs, and then patted dry to remove any residual isotonic solution. To determine fertilization rates, 50 μl sperm-extender solutions (salmon or trout) were placed on one side of a dry 1 L beaker. In a separate beaker, 1 mL of either the female's own ovarian fluid (=conspecific ovarian fluid condition) or a trout female's ovarian fluid (=heterospecific ovarian fluid condition) was added to 100 mL of Imsa river water, and this solution was then added immediately to the 1 L beaker containing the sperm-extender to initiate sperm activation and mixing. Within 1 sec of sperm activation, the washed eggs in the sieve were dipped into the activated sperm: river water solution for either 2, 5, or 10 sec. At the end of these gamete exposure times, the eggs were removed and passed rapidly through three solutions of clear river water to wash away any active sperm adhering to the egg membranes. Eggs were then placed in incubators and fertilization success scored 15 days later using acetic acid as described in *Noncompetitive fertilization experiments* above. Fertilization rate datasets showed no departures from normal distributions (Kolmogorov–Smirnov tests all *P* > 0.06), so, we used a repeated measures analysis of variance (ANOVA; to compare across related egg batches within females) to compare the relative variance in fertilization success explained by the three fixed factors of male species identity (conspecific or heterospecific), ovarian fluid identity (conspecific and heterospecific), and the three gamete exposure times (2, 5, and 10 sec).

#### Sperm competition trials comparing conspecific versus heterospecific sperm success

To measure the fertilization success of conspecific versus heterospecific sperm under simultaneous competition, egg batches containing on average 70 eggs (range 52–87 per batch) were exposed to homogenized mixes of 20 μl salmon and 20 μl trout sperm-extender solutions in 100 mL Imsa water. We employed a paired experimental design where gametes were split from individual fish (Yeates et al. 2009), so that an individual male's relative fertilization success could be compared in competition (against a male of the other species) for eggs from a conspecific versus a heterospecific female. This design therefore enabled control of any among-male variation in sperm competitiveness, and allowed us to isolate the variance in differential fertilization success that arose from cryptic female choice. Sixteen paired competitions were performed (using *n* = 32 different males) using eggs from *n* = 16 salmon females and then *n* = 16 trout females. To avoid pseudoreplication within sperm competition analyses (because of interdependence between competing pairs of males), we first analyzed from only the salmon male perspective, comparing sperm competition success of *n* = 16 male salmon (against *n* = 16 male trout) when they were either competing for salmon or trout eggs (all from different females). Thus, when competing for salmon eggs, the focal male here is a pure conspecific competitor, and when competing for trout eggs, he is a hybridizing heterospecific male competitor. We then repeated the analysis from the reciprocal trout male focal perspective using further paired comparisons; although this second analysis is not statistically independent of the first analysis (because the same competing pairs of salmon–trout males are being reanalyzed), this approach allowed us to check for any directional bias or asymmetry for either species in overall sperm competition outcome. Eggs were then allowed to develop for 2 months, after which a randomly selected subset of eyed embryos were preserved in ethanol for genetic analysis. An average of 27 offspring were genotyped to assign paternity in each fertilization trial (range 8–32). Fertilization datasets did not all conform to normal distributions (Kolmogorov–Smirnov tests *P* < 0.05), so paired analyses on square root arcsine transformed data (which then showed normality) were employed.

#### Controlling for hybrid embryo viability

Because hybrid embryos could suffer differential mortality, it was necessary to establish that sperm precedence was not confounded by embryo failure (although this could not explain the ovarian fluid effect we found for the ovarian fluid results below). We therefore ran a series of paired comparisons where eggs from *n* = 11 salmon and *n* = 11 trout were fertilized by both *n* = 11 salmon and *n* = 11 trout sperm-extender solutions (100 μl in 500 mL river water), and then measured the number of embryos still successfully developing after 3 months at the eyed stage (within 1–3 weeks of hatch). An average of 679 (±20 SE) eggs were used for each fertilization, and embryo development was measured as the difference between the number of eggs initially fertilized, and the number of embryos visible 3 months later. Embryogenesis success rate datasets showed no departures from a normal distribution (Kolmogorov–Smirnov tests all *P* > 0.06), so two paired *t-*tests were used to compare success of pure versus hybrid eggs in either species.

#### Measuring influences of egg and ovarian fluid identity on sperm competition success

To isolate the influence of ovarian fluid on CSP, a further set of in vitro sperm competition trials were conducted where salmon and trout eggs were exposed to homogenized mixes of salmon and trout sperm (as above), this time in the presence of either conspecific or heterospecific ovarian fluid. To separate eggs from their ovarian fluid identity, strips from ripe females were sieved just prior to fertilization trials, and ovarian fluid collected from each in separate beakers. Eggs in the sieve were then washed in an isotonic solution (90 g NaCl in 10 L of Imsa river water) to rinse away any remaining fluid from the surface of the eggs, and then patted dry to remove any residual isotonic solution. The egg batch of each female was then divided into two, and each placed on one side of a dry 1 L beaker. One milliliter of their own ovarian fluid was then pipetted over the eggs in one of the beakers (=conspecific ovarian fluid treatment), and 1 mL of ovarian fluid from a female of the other species was pipetted onto the eggs in the other beaker (=heterospecific ovarian fluid treatment). Sperm competitions were then run as described above using homogenized mixes of 20 μl salmon and 20 μl trout sperm-extender solutions, activated simultaneously by 100 mL Imsa river water.

The additional ovarian fluid treatment therefore created four competitive cross-combinations for each species: (1) salmon eggs in salmon ovarian fluid × salmon ♂ + trout ♂; (2) salmon eggs in trout ovarian fluid × salmon ♂ + trout ♂; (3) trout eggs in trout ovarian fluid × salmon ♂ + trout ♂; and (4) trout eggs in salmon ovarian fluid × salmon ♂ + trout ♂. Each of these combinations were replicated in 15 sperm competition trials using *n* = 15 ♀ salmon, *n* = 15 ♂ salmon, *n* = ♀ trout, and *n* = 15 ♂ trout. Each fertilization trial competed sperm for an average of 77 eggs (range 44–108). This paired factorial design allowed replicated comparisons of differential fertilization success of sperm from the same pair of competing males for conspecific or heterospecific eggs in either conspecific or heterospecific ovarian fluid. Fertilization success datasets showed no departures from normal distributions (Kolmogorov–Smirnov tests all *P* > 0.06). Results were analyzed using repeated measures ANOVA (to allow for cross-comparison within males) with egg identity and ovarian fluid identity as fixed factors. Again, because of interdependence between competing pairs of males, we analyzed first only from the salmon male perspective, comparing sperm competition success of *n* = 15 male salmon (competing with *n* = 15 male trout) when they were either competing for salmon or trout eggs in either salmon or trout ovarian fluid (from *n* = 15 + 15 different females). Thus, when competing for salmon eggs, the focal male is a pure conspecific competitor, and when competing for trout eggs, he is a hybridizing heterospecific male competitor, with both competitive scenarios taking place in either conspecific salmon or heterospecific trout ovarian fluid (each trial using different females). We then repeated the analysis from the reciprocal trout male focal perspective using a second repeated measures ANOVA; although this second analysis is not independent of the previous analysis, this approach allowed us to check for any directional bias or asymmetry for either species in the pattern of sperm competitiveness. Eggs from these trials were reared for 2 months, after which a random subset of eyed embryos were preserved in ethanol for genetic analysis. An average of 21 offspring were genotyped in each fertilization trial (range 13–26).

### PATERNITY ASSIGNMENT

DNA was extracted from adult fin clip tissue and offspring embryo tissue using a modified salt extraction technique (Aljanabi and Martinez 1997) in 96-well plates (ABgene, Surrey, U.K.). Paternity was assigned to offspring using up to three noninterrupted microsatellite loci: *Ssa408*, *ssa410*, and *Ssa417* (Cairney et al. 2000). The loci used were chosen as they amplify and exhibit substantial polymorphism in both Atlantic salmon and brown trout (Aljanabi and Martinez 1997; Yeates 2005). Once parental genotypes were known, often only a single locus was needed to unambiguously assign paternity in each two-male competition involving Atlantic salmon and brown trout. Polymerase chain reaction (PCR) was carried out in 10 volume reaction multiplexes containing: 1 μl of DNA (unspecified concentration), 5 μl of 2 × PCR Mastermix with 1.5 mM MgCl_2_ (ABgene), 0.95 μl of forward labeled primers (0.2 *Ssa408*, 0.3 *Ssa417*, and 0.45 *Ssa410*), and 0.95 μl reverse primers (same volumes). Primers were labeled with NED (*Ssa408*), FAM (*Ssa410*), and HEX (*Ssa417*; Applied Biosystems, Foster City, CA). The PCR ran with an initial 3 min denaturation at 94°C preceding 29 denaturing (94°C for 15 sec), annealing (61°C for 15 sec), and extension (72°C for 15 sec) cycles. Samples were finally incubated at 72°C for 30 min. Polymerase chain reaction products were run on an ABI3730 automated sequencer at the NERC Biomolecular Analysis Facility at the University of Sheffield. Samples were run with Genescan-500 ROX labeled size standard (Applied Biosystems Foster City, California). Fragment lengths of PCR products were determined using the genotyping software GeneMapper v4.0 (Applied Biosystems Foster City, California).

### SPERM BEHAVIOR ANALYSES

To measure the influence of ovarian fluid on sperm activity, we employed Computer Assisted Sperm Analysis (CASA) optimized for fish (Kime et al. 2001) to compare behavior of sperm activated in river water versus both species’ ovarian fluid for *N* = 16 salmon and 15 trout. Sperm-extender solutions were examined within 24 h of strip, and activated in either river or ovarian fluid, then 0.7 μl of the activated diluent rapidly transferred onto a 12-well multitest glass slide (ICN Basingstoke, U.K.; well depth 0.0116 mm) and a round cover slip immediately put in place (Yeates 2005). Sperm activity was recorded onto Sony Hi8 videotapes from a JVC video camera (TK-1280E) fixed to an Olympus CK40 inverted stage microscope at ×400 under dark field phase illumination. The volume ratio of sperm-extender to activation solution (water or ovarian fluid) was adjusted so that 50–100 spermatozoa were visible in the field of view at 400× magnification for each trial (Gage et al. 2004; Yeates 2005). To eliminate sperm motility variance due to water temperature, all activations and recordings were performed in a cold room at 6.5°C. Using CASA, we measured: (1) %motility (=the proportion of visible sperm showing forward progression), (2) curvilinear velocity (=average sperm swimming speed: the average speed of progression along sperm swimming paths), (3) longevity (=the active life span of the sperm sample, measured manually as the time at which all sperm visible in the field of view ceased forward swimming progression), and (4) linearity or straightness (=sperm swimming trajectories, measured as the average proportion derived from the ratio between the total trajectory distance swum versus the straight-line distance between the start and end of the path, and where perfect straightness = 1.0 [Kime et al. 2001; Yeates 2005]). Sperm motility was measured through analysis of the Hi8 videotapes by CASA using a Hobson Sperm Tracker (Hobson Vision Ltd., Baslow, U.K.) at the Zoological Society of London. Salmonid sperm typically show rapid swimming velocity over a brief life span (under 30–60 sec; Yeates 2005; Yeates et al. 2007), so tracking data on %motility, curvilinear velocity, and path straightness were collected for 15 sec from 10 sec after the time of sample activation (Kime et al. 2001). Longevity was the period from activation until sperm ceased forward progressive motility. The Hobson tracker was set to operate at a frame rate of 50 Hz and the “minimum track point” setting was 50 frames. The “search radius” used was 8.13–10.56 μm and the “threshold” set to +30/−100 with the objective at 40×. To represent differences in swimming behavior, paths of salmon sperm swimming in river water and salmon ovarian fluid were plotted using head positions at 0.05 sec intervals across the field of view to construct 1 sec continuous tracks. Tracks were plotted for samples within 5 sec of activation, and only those tracks plotted, which began in the field of view and swam for the majority of their path within the field of view. The two movies from which these tracks were constructed are available in Supporting Information (Videos S1 and S2). None of the sperm motility datasets departed from a normal distribution (Kolmogorov–Smirnov tests all *P* > 0.06), so differences between the three treatments (activation in river water, conspecific, and heterospecific ovarian fluid) were analyzed using a linear model with treatments as fixed factors; to take advantage of the factorial paired design (where sperm from individual males were assayed in each of the three treatments), we included male identity as a random factor in the model. Models indicating significant variances between treatments were then analyzed using post hoc Tukey tests to identify where differences existed.

### IN VITRO SPERM MIGRATION ASSAYS

A modified Corning-Costar Transwell® cell migration assay was employed to measure the dispersal of water-activated salmon and trout sperm through a porous membrane into ovarian fluid (Olson et al. 2001). We used Transwells® with inserts containing a 10-μm thick polycarbonate basal membrane permeated by 8-μm diameter pores at a density of 1 × 10^5^ cm^2^ (Corning Life Sciences, Tewksbury, MA). Micropyles of *Oncorhynchus* salmon and trout eggs have diameters between 15 and 40 μm across the entrance vestibule, narrowing to between 2 and 4 μm across the canal (Yanagimachi et al. 1992), so an 8-μm pore diameter provides a relevant compromise dimension. Two-hundred microliters of trout or salmon ovarian fluid (plus a river water control) was placed in the outer well, and 50 μl of river water in the inner well. Twenty microliters of sperm-extender was then pipetted into the river water within the inner well and activated. After 2 min, the inner well was removed and any residual fluid attached to the basal membrane was washed off with a further 500 μl of water. The fluid in the outer well, now containing ovarian fluid, water, and any migrated sperm cells, was then mixed and pipetted into microcentrifuge tubes for counting. Numbers of sperm that had traversed the porous membrane were then counted using improved Neubauer hemocytometers (Gage et al. 2004; Yeates 2005). Dispersion of sperm from *n* = 18 male salmon and *n* = 17 male trout were tested in Transwells® containing either salmon ovarian fluid, trout ovarian fluid, or river water. All sperm migration trials were conducted over a single day in a walk-in fridge at 6.5°C to mimic natural spawning water temperatures. Sperm migration datasets showed significant departures from normality, even after transformation, so we applied nonparametric testing. Results from this factorial experimental design were analyzed using a nonparametric Friedman test to compare dispersal of related samples (sperm from individual males) in three different treatment conditions: (1) conspecific and (2) heterospecific ovarian fluid, and (3) river water.

## Results

### FERTILIZATION COMPATIBILITY BETWEEN SPECIES WITHOUT SPERM COMPETITION

**Figure 1:**
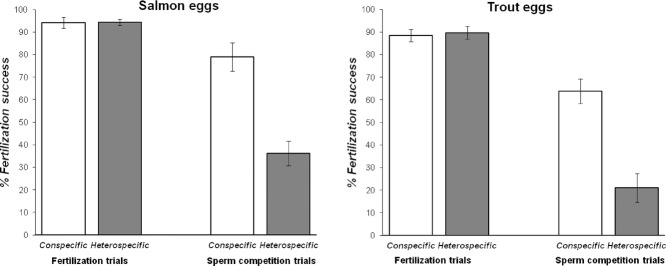
Differential fertilization compatibility between salmon and trout sperm and eggs in the absence or presence of opportunity for cryptic female choice. Bars showing mean %fertilization success (±SE) for either salmon or trout eggs when exposed to conspecific (white bars) or heterospecific (gray bars) sperm. Fertilization trials using gametes from one female and one male (*n* = 15 paired replicates per treatment) provided no opportunity for cryptic female choice, and no differences in relative fertilization success were found for eggs from either salmon (*t*_14_ = 0.47, *P* = 0.65) or trout (*t*_14_ = −0.805, *P* = 0.43). Sperm competition trials (*N* = 16 paired replicates per treatment) exposed eggs to conspecific and heterospecific sperm simultaneously, providing opportunity for cryptic female choice, and revealing significant conspecific sperm precedence in eggs of both salmon and trout (*t*_15_ = 7.19, *P* < 0.001, for both species).

**Figure 2:**
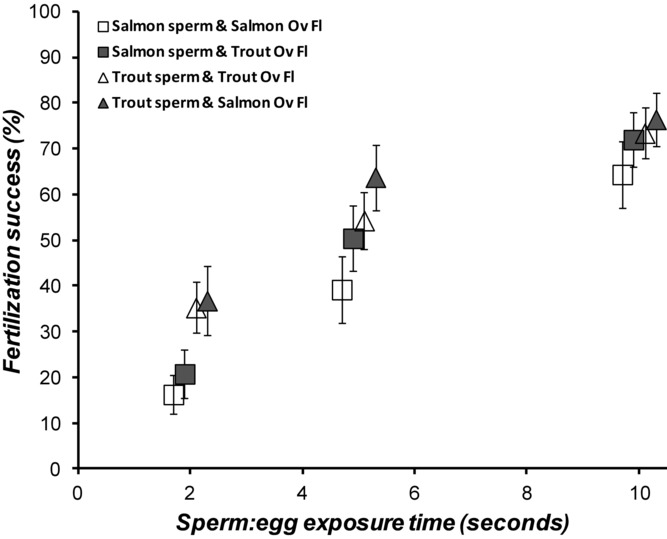
Fertilization rates of salmon eggs decline with limited sperm exposure time, but are unaffected by species identities of sperm or ovarian fluid. Means (±SE) are fertilization success of *n* = 15 trials per treatment of salmon eggs exposed for 2, 5, or 10 sec to either salmon sperm (squares) or trout sperm (triangles) in the presence of either conspecific salmon (clear markers) or heterospecific trout ovarian fluid (gray markers). Using a repeated measures ANOVA (to compare across related egg batches within females), we found that only gamete exposure time showed significant difference between treatments (*F*_1,14_ = 58.5, *P* < 0.001), and no significant effect of male species identity (*F*_1,14_ = 2.9, *P* = 0.111), or ovarian fluid identity (*F*_1,14_ = 0.669, *P* = 0.427).

Using noncompetitive in vitro fertilization experiments, where a single male's sperm were added to a single female's eggs, we found complete interfertility at the gamete level between salmon and trout gametes (Fig. 1: fertilization trials comparisons). Even when we limited the opportunity for sperm to access the egg micropyle to a few seconds by washing away activated sperm from eggs after 2, 5, or 10 sec following the start of in vitro fertilization, we found no difference between fertilization rates of salmon ova with either conspecific or heterospecific sperm (Fig. 2). Thus, we found no fertilization barriers preventing hybridization between salmon and trout sperm and eggs.

### FERTILIZATION COMPATIBILITY BETWEEN SPECIES WITH SPERM COMPETITION

When we provided ova under similar fertilization conditions with homogenous mixes of identical quantities of both species’ sperm in competition for fertilizations, we discovered clear evidence for CSP (Fig. 1). Because our experimental crossing design was factorial and paired, comparing the variance in differential fertilization success of sperm from individual males in both conspecific and heterospecific fertilizing roles (and not therefore confounded by intermale variation in sperm quality), and because we removed whole animal effects, we can therefore conclude that this CSP is due to cryptic female choice by both species’ eggs for the most genetically compatible sperm. Thus, salmon and trout eggs, when provided with a simultaneous choice of sperm from conspecific and heterospecific males, constrained average fertilization success of heterospecific sperm between 20% and 35%, with a relatively symmetrical preference for conspecific sperm by both species’ eggs.

To be sure that this CSP was not confounded by differential embryo mortality, we ran parallel assays of embryo development (within 1–3 weeks of hatch) and found no differences in embryogenesis success between pure and hybrid eggs that could confound our sperm precedence findings. Salmon eggs fertilized by salmon sperm showed equivalent embryogenesis success rates as those fertilized by trout sperm (*t*_10_ = −1.007, *P* = 0.34; ♀ salmon × ♂ salmon = 75.0 [±6.1]% success, ♀ salmon × ♂ trout = 79.5 [±5.8]% success). Trout eggs fertilized by trout sperm showed a higher rate of hatch than those fertilized by salmon sperm (*t*_10_ = 2.216, *P* = 0.051; ♀ trout × ♂ trout = 73.8 [±5.0]% success, ♀ trout × ♂ salmon = 66.59 [±5.8]% success), but the difference was nonsignificant with a 7.2% difference in embryo development success. Together with the salmon egg hybrid embryo development success rates, which were 4.5% higher in hybrid crosses, these variations in embryo development success rates cannot explain the >40% differences we found in sperm precedence rates between conspecific and heterospecific males for both salmon and trout, so we can be confident that differential hybrid embryo survival does not confound our sperm precedence findings (Fig. 1: sperm competition trials).

### OVARIAN FLUID INFLUENCE ON CSP

**Figure 3:**
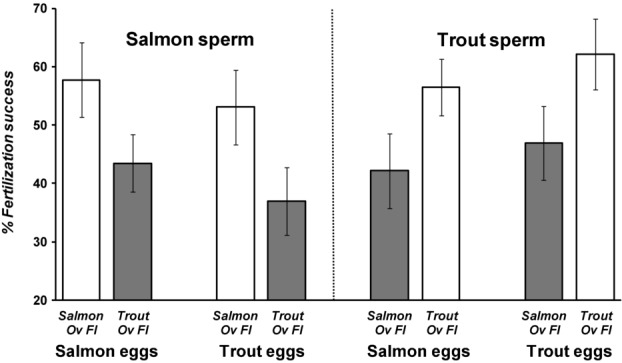
Ovarian fluid controls the patterns of conspecific sperm precedence in salmon and trout in vitro fertilization competitions. Average sperm competition success (±SE) for male salmon–trout pairs (*n* = 15 paired replicates per treatment) competing for salmon or trout eggs in conspecific (white bars) or heterospecific (gray bars) ovarian fluid. Differential fertilization success was most strongly influenced by the presence of conspecific versus heterospecific ovarian fluid (salmon: *F*_1,14_ = 6.62, *P* = 0.022; trout: *F*_1,14_ = 6.14, *P* = 0.027), whereas the species identity of the eggs had a nonsignificant influence (salmon: *F*_1,14_ = 4.57, *P* = 0.051; trout: *F*_1,14_ = 3.35, *P* = 0.089) and there was no interaction effect of egg and ovarian fluid identity (salmon: *F*_1,14_ = 0.117, *P* = 0.737; trout: *F*_1,14_ = 0.031, *P* = 0.863).

In noncompetitive fertilizations, with no opportunity for cryptic female choice, we again found complete interfertility between salmon eggs and both salmon and trout sperm, and this was not affected by the presence of conspecific or heterospecific ovarian fluid (Fig. 2). Even when we limited sperm exposure to eggs for as little as 2 sec, we found no difference in the relative fertilities of conspecific and heterospecific sperm, in either conspecific and heterospecific ovarian fluid (Fig. 2). However, when we invoked sperm competition, by providing eggs with homogenized mixes containing equal volumes of both salmon and trout sperm, we again identified clear evidence in both species of cryptic female choice, except in this experiment, we were able to identify that the fertilization biases were dependent upon the presence of conspecific ovarian fluid, and not a function of egg identity (Fig. 3). Thus, we found that CSP was mediated by the presence of conspecific ovarian fluid in both salmon and trout, with the species’ identify of the egg playing minor, nonsignificant roles.

### OVARIAN FLUID INFLUENCE ON SPERM BEHAVIOR

**Figure 4:**
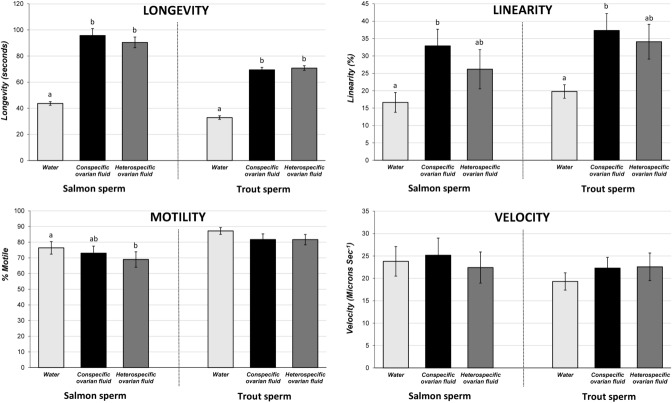
Mean CASA measures (±SE) of sperm behavior in river water (white bars) versus conspecific (black bars) or heterospecific ovarian fluid (gray bars) for *N* = 16 male salmon and 15 male trout. In both species, ovarian fluid increased sperm motile life span (LONGEVITY; salmon: *F*_2,30_ = 65.33, *P* < 0.0001; trout: *F*_2,28_ = 212.5, *P* < 0.0001) and straightened swimming trajectory (LINEARITY; salmon: F_2,30_ = 3.45, *P* = 0.045; trout: *F*_2,28_ = 4.33, *P* = 0.023). Letters above bars that are different identify where Tukey tests find significant post hoc differences at *P* < 0.05, revealing evidence for species-specific effects of ovarian fluid on sperm linearity in both salmon and trout (see Results for further details). Ovarian fluid did not influence sperm swimming velocity (VELOCITY; salmon: *F*_2,30_ = 0.143, *P* = 0.87; trout: *F*_2,28_ = 0.68, *P* = 0.52). There were also no effects of ovarian fluid on the proportions of sperm that were progressively motile in trout (*F*_2,28_ = 2.23, *P* = 0.127) but, in salmon, heterospecific ovarian fluid caused a marginal decrease in the proportion of motile sperm, relative to water (*F*_2,30_ = 3.55, *P* = 0.041).

**Figure 5:**
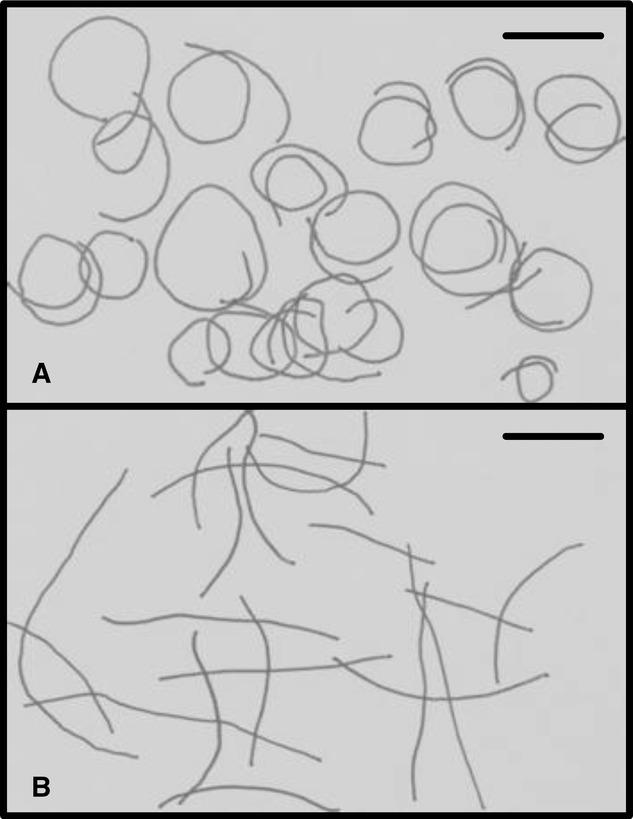
Representative salmon sperm swimming tracks show a straightening of path trajectory from water (A) to ovarian fluid (B). Each track traces 1 sec of sperm movement, at 5 sec after initial activation. Tracks are reconstructed from drawings of sperm head positions plotted every 0.05 sec. Videos are available online in Supplementary Information. Scale bars are 25 μm.

CASA (Kime et al. 2001) measures of sperm behavior revealed that both salmon and trout sperm had more than twice the motile life span, and followed significantly straighter swimming trajectories in ovarian fluid, compared with river water (Figs. 4 and 5, and online Supporting Information Videos S1 and S2). Straightening of the sperm swimming path is a possible mechanism of chemoattraction by the ovum (Ward et al. 1985), and linearity was elevated in ovarian fluid (compared with water) in both salmon (*F*_2,30_ = 3.45, *P* = 0.045) and trout (*F*_2,28_ = 4.33, *P* = 0.023). In both species, post hoc Tukey testing revealed that significant changes in linearity only occurred in conspecific ovarian fluid (water vs conspecific ovarian fluid: salmon *P* = 0.036, trout *P* = 0.026), and not in heterospecific ovarian fluid (water vs heterospecific ovarian fluid: salmon *P* = 0.291, trout *P* = 0.08; Fig. 4). In addition to changes in linearity, ovarian fluid allowed a longer progressive life span for both species’ sperm (salmon: *F*_2,30_ = 65.33, *P* < 0.0001; trout: *F*_2,28_ = 212.5, *P* < 0.0001). This change in longevity was not specific to conspecific ovarian fluid, however, and post hoc testing revealed that both species showed significant differences between longevity in water versus both conspecific and heterospecific ovarian fluid (all four Tukey tests *P* < 0.0001; Fig. 4). We found no changes in sperm curvilinear swimming velocity between water and ovarian fluid for either salmon (*F*_2,30_ = 0.143, *P* = 0.87) or trout (*F*_2,28_ = 0.68, *P* = 0.52; Fig. 4). There were also no effects of ovarian fluid on the proportions of sperm that were progressively motile in trout (*F*_2,28_ = 2.23, *P* = 0.127), but in salmon the heterospecific ovarian fluid caused a marginal decrease in the proportion of motile sperm, relative to water (*F*_2,30_ = 3.55, *P* = 0.041, Fig. 4).

### SPERM CHEMOATTRACTION BY OVARIAN FLUID

**Figure 6:**
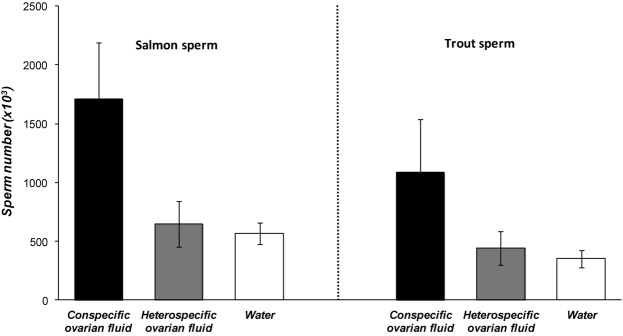
Conspecific ovarian fluid attracts more sperm. Mean numbers (±SE) of activated salmon (*n* = 18 males) and trout (*n* = 17 males) sperm passing through 8-μm diameter Transwell® membrane pores into conspecific ovarian fluid (black bars), heterospecific ovarian fluid (gray bars), and river water (white bars). Significantly greater numbers of sperm passed through into conspecific ovarian fluid compared with heterospecific ovarian fluid and water (salmon: Friedman test: χ^2^ = 12.38 (2df), *P* = 0.002; trout: Friedman test: χ^2^ = 15.08 (2df), *P* = 0.001).

Our modified Transwell® cell migration assay confirmed that conspecific ovarian fluid could act as a chemoattractant to the ovum through 8-μm diameter pores, mimicking the size of the salmonid egg micropyle (Yanagimachi et al. 1992). We found a significantly greater number of sperm traversed the Transwell® membranes into a solution of their own conspecific ovarian fluid, compared with either heterospecific fluid or river water (Fig. 6).

## Discussion

Through a series of controlled experiments at the gamete level, we were able to show that salmon and trout gametes, despite having complete potential interfertility, exhibit a fertilization preference by eggs for conspecific sperm if provided with a choice. We found that this preference for conspecific sperm is controlled by ovarian fluid, because the presence of conspecific ovarian fluid around the eggs during fertilization was necessary to allow CSP. Remarkably, we discovered that the biology and species identity of the egg had, at best, a secondary influence because it was only when conspecific ovarian fluid was present that conspecific precedence could be achieved and, moreover, we were able to give heterospecific sperm a competition advantage within hybrid fertilizations by adding their own species’ ovarian fluid to the fertilization mix. CASA (Kime et al. 2001) assays showed that conspecific ovarian fluid encouraged a much straighter sperm swimming path, and ovarian fluid of either species allowed a longer life span compared with river water (the change in swimming trajectory is evident from the Supporting Information Videos available online). Our final experiment to measure the in vitro chemoattractive properties of conspecific ovarian fluid confirmed a species-specific attraction for conspecific sperm in traversing a Transwell® membrane permeated by pores that mimicked the size of the salmonid egg micropyle (Yanagimachi et al. 1992). Our sperm behavior, fertilization, and sperm competition results combined indicate that ovarian fluid allows more effective chemoattraction of conspecific sperm toward the vestibule and down the micropyle to fertilize, most likely by switching sperm behavior using fast-evolving, species-specific gamete recognition proteins (Vacquier 1998; Swanson and Vacquier 2002; Palumbi 1999) to follow a straighter swimming path over a longer life span. One mechanism of sperm chemoattraction is through the straightening of a previously elliptical swimming trajectory, which allows directed chemotaxis into and up a biochemical concentration gradient (Ward et al. 1985). If the sperm remains within the concentration, it continues its straight trajectory, however, if it exits, the elliptical swimming pattern can be reinstated, encouraging a pathway that returns into the concentration gradient, and therefore back on target (Kashikar et al. 2012).

Because our experimental designs were factorial and paired throughout, our findings are free of the known interindividual variation in gamete quality (e.g., Gage et al. 2004), and therefore the result of interactions and compatibilities between male and female gametes that constitute cryptic female choice. We can therefore identify ovarian fluid as the factor that allows cryptic choice of conspecific sperm, but it is important to stress that our experiments also reveal that this choice only occurs in the context of sperm competition. The findings combined therefore exemplify that sperm competition and cryptic female choice should not be viewed as dichotomous phenomena, but processes that clearly interact within the sperm–egg arena where postcopulatory sexual selection operates. We have deliberately chosen an external fertilization system to experimentally tease apart the interacting roles of sperm, egg, and ovarian fluid in the struggle to fertilize and reproduce, but a glance at the literature on sperm and female tract functional diversity (Birkhead et al. 2009) indicates an exponential jump up in the complexity of interactions proceeding within internal fertilization systems; there is therefore much to discover within postcopulatory sexual selection.

In the absence of sperm choice, we find no effective barriers to hybridization between sperm and egg in salmon and trout (Fig. 1 and 2), reinforcing the relevance of considering postcopulatory mechanisms of sperm competition and cryptic female choice for understanding reproductive isolation between species. The salmon mating pattern is known for its high levels of sperm competition (Fleming 1996), with a recent molecular study of natural paternity levels revealing that an average of eight males, and up to 16, are successfully involved in simultaneous competition to fertilize a single nest (Weir et al. 2010). There is thus intense sperm competition occurring over the very brief timescale, while eggs are fertilizable and released into each nest (Gage et al. 2004). It is conceivable that this high level of female promiscuity could be promoted by the risks of genetic incompatibility between males and females within a population (Michalczyk et al. 2011).

Our findings reveal an important relationship between signals contained within ovarian fluid and sperm function. Ovarian fluid comprises 10–30% of the total egg mass volume, bathes ovulated eggs in the female's peritoneum, and is released at spawning around the eggs (Rosengrave et al. 2009). Ovarian fluid is known to influence sperm swimming behavior in fish (Turner and Montgomerie 2002; Rosengrave et al. 2009), either enhancing (Butts et al. 2012) or slowing (Gasparini and Pilastro 2011) sperm movement according to relatedness, or the male–female combination (Rosengrave et al. 2008), and explaining reduced sperm competition success when mating with sisters in guppies (Gasparini and Pilastro 2011). This latter study in internally fertilizing guppies shows that ovarian fluid may also allow avoidance of gamete incompatibility presenting risks of inbreeding, as well as the avoidance of outbreeding through fertilization by unrelated haplotypes as we find here. Because ovulated eggs are bathed in ovarian fluid within the coelomic cavity of female fishes, concentrations during spawning are likely to be high close to the egg outer membrane, and highest inside the single egg micropyle and vestibule, into which the successful sperm must swim to access the ooplasm (Yanagimachi et al. 1992). Analyses of activated sperm in different fish species (including salmonids) show that the micropyle and vestibule have chemoattractant properties to spermatozoa, which are species-specific in marine spawning black, barfin, and starry flounders (Yanagimachi et al. 2013). Because the straightening of a previously elliptical swimming trajectory allows directed chemotaxis into and up a biochemical concentration gradient (Ward et al. 1985), if ovarian fluid is most concentrated inside the micropyle, which seems probable, then we propose this as the mechanism that allows cryptic female choice of conspecific sperm in natural spawnings. Fast-evolving reproductive proteins (Vacquier 1998; Swanson and Vacquier 2002; Palumbi 1999) are likely candidates to allow species-specific signaling between ovarian fluid and sperm, switching the behavior of conspecific sperm via changes in ion channels that modify flagellar beat and therefore swimming direction toward the “right” egg (Kaupp et al. 2003).

We selected hybridization between salmon and trout as our system to test for evidence of cryptic female choice because (1) we could control for intermale confounding effects using split-brood and split-ejaculate in vitro fertilization experiments; (2) the ability to assay natural sperm behavior was present under external fertilization; and (3) there is clear a priori evidence from these systems that selection should act (especially on females) to avoid genetically incompatible heterospecific sperm. Our findings confirm CSP mediated by cryptic female choice as one mechanism to promote isolation between these sympatric species. However, the question remains as to whether this conspecific male–female reproductive compatibility is the result of drift in the coevolutionary mechanisms of sperm–egg association within either species, perhaps facilitated by male:female sexual antagonism (Martin and Hosken 2003), or whether reinforcement against risks of hybridization within sympatry have led to a promotion of incompatibility between the two species where they coexist (Coyne and Orr 2004). Certainly, within-species differences in interpopulation compatibility can evolve, as exemplified by consubspecific sperm precedence in *Drosophila pseudoobscura* (Dixon et al. 2003). There is also some evidence for reinforcement of such male:female incompatibilities under heightened risks of hybridization: in *Drosophila yakuba*, which can hybridize with *D. santomea* in Sao Tome: females from within the hybrid zone demonstrate increased gametic isolation from *D*. *santomea* compared with females experiencing lower risks of hybridization outside the zone (Matute 2010). However, there is also countering evidence that reinforcement promotes gametic incompatibilities: recent analyses of bindin divergence within the *Arbacia* urchin genus provide no evidence that reinforcement has driven elevated change (Lessios et al. 2012), and comparisons of in vitro fertilization rates between two potentially hybridizing *Mytilus* species found that populations in sympatry were actually more interfertile than populations in allopatry (Slaughter et al. 2008). There is clearly opportunity for further work here and we plan to measure whether the levels of CSP, we find here, are repeatable across salmon–trout crosses that exist under varying levels of isolated allopatry.

## Supporting Information

Sperm motility videos are linked to the online version of the paper at: http://onlinelibrary.wiley.com/doi/10.1111/evo.12208/suppinfo. Representative movies showing 6.7 sec of activated salmon sperm at ×400 under dark field phase contrast commencing 3–5 sec following activation in salmon ovarian fluid (*Video 1*) and river water (*Video 2*). Sperm trajectories follow a straighter path in ovarian fluid (*Video 1*) compared with circular paths in water (*Video 2*).
